# Beta_1_-Adrenoceptor Polymorphism Predicts Flecainide Action in Patients with Atrial Fibrillation

**DOI:** 10.1371/journal.pone.0011421

**Published:** 2010-07-02

**Authors:** Amir M. Nia, Evren Caglayan, Natig Gassanov, Tom Zimmermann, Orhan Aslan, Martin Hellmich, Firat Duru, Erland Erdmann, Stephan Rosenkranz, Fikret Er

**Affiliations:** 1 Department of Internal Medicine III, University of Cologne, Cologne, Germany; 2 Institute of Medical Statistics, Informatics and Epidemiology, University of Cologne, Cologne, Germany; 3 Clinic for Cardiology, University Hospital Zurich, Zurich, Switzerland; University of Illinois at Chicago, United States of America

## Abstract

**Background:**

Antiarrhythmic action of flecainide is based on sodium channel blockade. Beta_1_-adrenoceptor (β_1_AR) activation induces sodium channel inhibition, too. The aim of the present study was to evaluate the impact of different β_1_AR genotypes on antiarrhythmic action of flecainide in patients with structural heart disease and atrial fibrillation.

**Methodology/Principal Findings:**

In 145 subjects, 87 with atrial fibrillation, genotyping was performed to identify the individual β_1_AR Arg389Gly and Ser49Gly polymorphism. Resting heart rate during atrial fibrillation and success of flecainide-induced cardioversion were correlated with β_1_AR genotype. The overall cardioversion rate with flecainide was 39%. The Arg389Arg genotype was associated with the highest cardioversion rate (55.5%; OR 3.30; 95% CI; 1.34–8.13; *p* = 0.003) compared to patients with Arg389Gly (29.5%; OR 0.44; 95% CI; 0.18–1.06; *p* = 0.066) and Gly389Gly (14%; OR 0.24; 95% CI 0.03–2.07; *p* = 0.17) variants. The single Ser49Gly polymorphism did not influence the conversion rate. In combination, patients with Arg389Gly-Ser49Gly genotype displayed the lowest conversion rate with 20.8% (OR 0.31; 95% CI; 0.10–0.93; *p* = 0.03). In patients with Arg389Arg variants the heart rate during atrial fibrillation was significantly higher (110±2.7 bpm; *p* = 0.03 vs. other variants) compared to Arg389Gly (104.8±2.4 bpm) and Gly389Gly (96.9±5.8 bpm) carriers. The Arg389Gly-Ser49Gly genotype was more common in patients with atrial fibrillation compared to patients without atrial fibrillation (27.6% vs. 5.2%; HR 6.98; 95% CI; 1.99–24.46; *p*<0.001).

**Conclusions:**

The β_1_AR Arg389Arg genotype is associated with increased flecainide potency and higher heart rate during atrial fibrillation. The Arg389Gly-Ser49Gly genotype might be of predictive value for atrial fibrillation.

## Introduction

Flecainide is used for cardioversion in patients with recent onset atrial fibrillation (AF). It has been successfully implemented for out-of-hospital use as a pill-in-the-pocket approach in patients without structural heart disease [1], [2]. In patients with organic heart disease our group recently reported that a single dosage of flecainide is not harmful, but less efficient than in healthy subjects [3]. Only few factors which might predict the success of flecainide in acute cardioversion have been identified, yet. The duration of AF and the extent of cardiac remodeling in diseased heart might be such factors.

The antiarrhythmic effect of flecainide is based on Na^+^ current (I_Na_) inhibition [4]. Experimental data have shown that beta_1_- adrenergic receptor (β_1_AR) stimulation causes an inhibition of I_Na_, too [5], [6], [7], [8].

For the β_1_AR, 12 nucleotide polymorphisms (SNPs) have been identified, but only two have being associated with possibly functional relevance (for review see [9]). At position 389 (P389), the putative stimulatory G-protein coupling domain of β_1_AR, glycine (Gly389) is substituted by arginine (Arg389). The Arg389 variants exhibited higher basal adenyl cyclase activity than the Gly389 variants [10], [11]. In clinical experiments patients with Arg389 allele exerted augmented cardiac contractile response to catecholamines [12]. The second potential relevant polymorphism is linked to the position 49 (P49) of β_1_AR, where serine (Ser49) is substituted by glycine (Gly49) [10], [13], [14]. Ser49Gly β_1_AR is considered to play a modulating role without affecting the agonist binding and basal or maximal stimulated adenyl cyclase activity [10], [15], [16].

In the present study we aimed to determine whether the two SNPs P389 and P49 of β_1_AR are relevant in predicting AF, antiarrhythmic drug therapy and flecainide-induced cardioversion in patients with recent onset AF.

## Methods

### Ethics Statement

The study complies with the Declaration of Helsinki, the local ethics committee of the University of Cologne has approved the research protocol. All patients gave their written informed consent.

### Subjects

**Figure 1:**
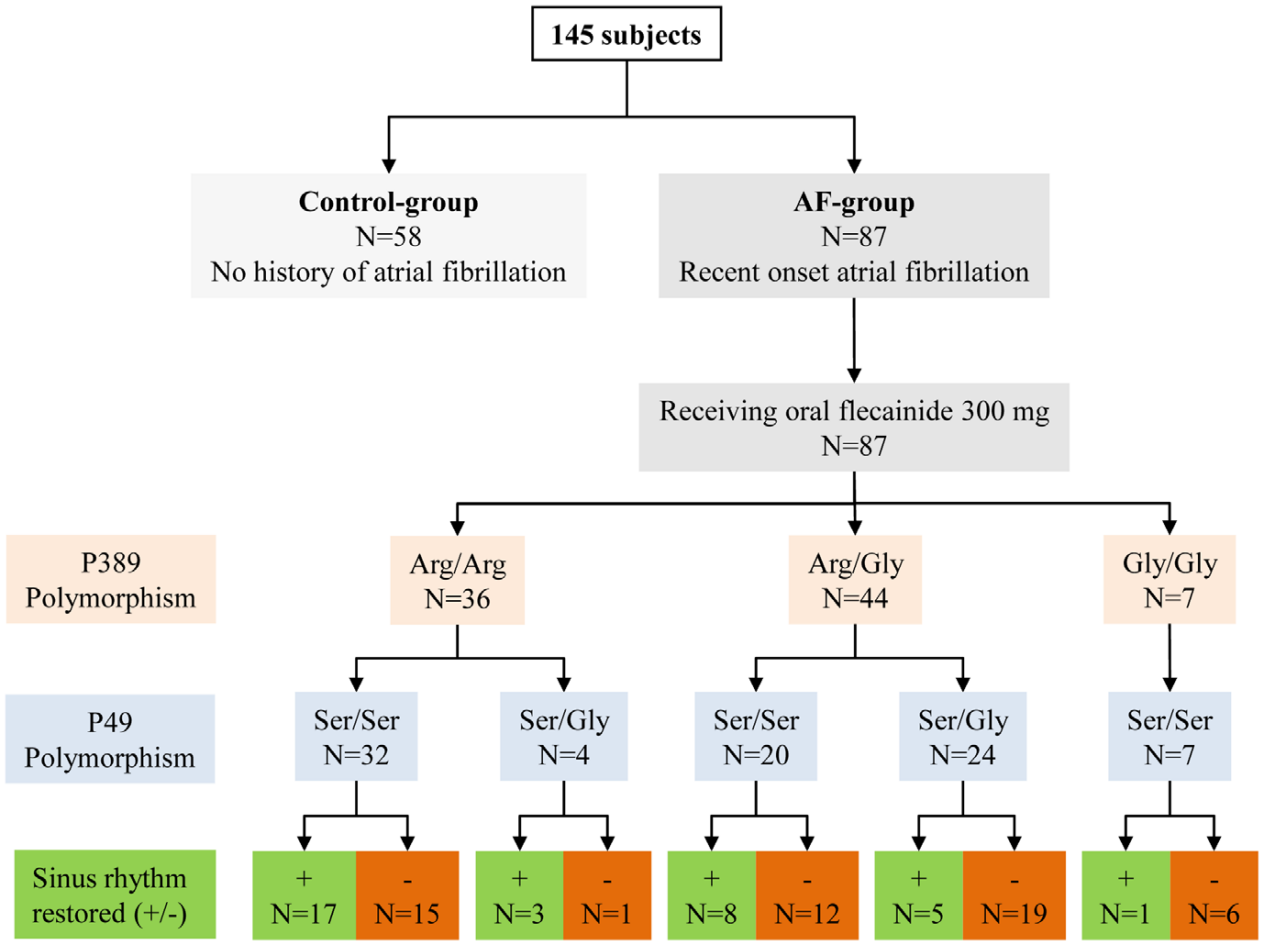
Study flow chart. Allele frequencies of different SNPs at position 389 and 49. The Gly49Gly variant was not present in any patient. All seven patients with Gly389Gly were homozygous for Ser49Ser.

The cohort of 145 subjects was composed of patients with recent onset (<10 days, mean 5.8±0.5 days) AF (AF-group; n = 87) and matched patients without documented and subjective history of AF (control group; n = 58; Figure 1). Patients with AF were at age above 18 years and revealed at least one of the additional comorbidities: coronary heart disease (CHD), dilated cardiomyopathy (DCM) with reduced left ventricular ejection fraction (<50%), heart failure (> NYHA II) or a PROCAM-score above 41 [17]. In all patients with AF transesophageal echocardiography was performed to exclude the presence of intracardiac thrombi. Routine transthoracic echocardiography was performed to measure cardiac dimensions and asses valvular, left and right ventricular function.

Matched patients in the control group were admitted to our hospital with acute myocardial infarction for percutaneous coronary intervention, without any history of AF.

### Sinus rhythm cardioversion

For pharmacological cardioversion patients with AF received oral flecainide 300 mg. A successful cardioversion was defined as sinus rhythm restoration within the first 6 hours after flecainide intake. After 12-h ECG monitoring had confirmed the persistence of a stable sinus rhythm. Patients in which flecainide did not restore sinus rhythm underwent electrical cardioversion (data not shown). At four weeks the persistence of sinus rhythm was controlled.

### Genotyping

Genomic DNA was extracted from blood of subjects by standard techniques [18]. Polymorphism detection was performed like previously described [10]. Briefly, DNA was amplified by polymerase chain reaction and subjected to enzymatic restriction analysis by *Bsm*F1 for the position 389 and *Eco01091* for the position 49 [10], [12]. Genotyping was performed blindly to all other data.

### Statistical analysis

All variables were tested for normal distribution with the Kolmogorov-Smirnov test. Continuous variables are expressed as means ± standard error of the mean. Comparison of 2 means was performed with the *t* test for normally distributed variables and the Mann-Whitney *U* test for non-Gaussian variables. Chi-square test was used for nonparametric comparisons. All statistical tests were 2-tailed, and *p*<0.05 was considered statistically significant.

## Results

### Patient Characteristics

The mean age of participants with AF was 67.8±1.3 years (71% men). Genotyping revealed a prevalence of homozygous patients for Arg389Arg of 41.4% (n = 36), heterozygous for Arg389Gly of 50.6% (n = 44) and homozygous for Gly389Gly of 8% (n = 7). 59 patients (67.8%) with AF were homozygous for serine at position 49 (Ser49Ser) and 28 patients (32.2%) heterozygous for Ser49Gly. The Gly49Gly genotype was not present in any patient (Figure 1). Baseline characteristics were similar in all variants (Table 1). In particular the calculated PROCAM-score, serum potassium and sodium concentrations were not significantly divergent between different allele carriers. Baseline heart rate during AF was significantly higher in patients with the Arg389Arg genotype with 110.7±2.7 bpm versus 104.8±2.4 bpm in patients with Arg389Gly and 96.9±5.8 bpm in Gly389Gly carriers (*p* = 0.03). In patients homozygous to Arg389Arg the heart rate was not influenced by Ser49Ser (n = 32; 110.8±3.0 bpm; *p* = ns) and Ser49Gly (n = 4; 110.0±4.1 bpm; *p* = ns). The resting heart rate was not modified in the same manner by Ser49Ser or Ser49Gly polymorphism in patients heterozygous for Arg389Gly (Ser49Ser: n = 20; 102.5±2.9 bpm; Ser49Gly: n = 24; 106.7±3.0 bpm; *p* = ns). In patients homozygous for Gly389Gly only the Ser49Ser variant was present.

**Table 1:** Baseline characteristics in patients with different β_1_AR variants.

	All patients with AF N = 87	Arg389Arg N = 36	Arg389Gly N = 44	Gly389Gly N = 7	Ser49Ser N = 59	Ser49Gly N = 28
**Women/Men (%)**	25/62 (29/71)	8/28 (22/78)	14/30 (31/69)	3/4 (43/57)	18/41 (31/29)	7/21 (25/75)
**Age**	67.8±1.3	67.1±2.1	67.7±1.8	72.0±2.6	67.1±2.1	69.3±3.8
**BMI**	26.6±0.4	27.2±0.5	26.4±0.5	24.8±1.0	26.4±0.7	27.0±0.5
**Heart rate per minute**	106.6±1.7	110.7±2.7*	104.8±2.4	96.9±5.8	107.4±1.8	104.9±2.4
**PROCAM-Score**	44.4±0.5	44.2±0.7	44.9±0.8	42.8±1.3	44.4±0.7	44.4±0.5
**Hypertension (%)**	47 (54)	17 (47)	25 (57)	5 (71)	33 (56)	14 (50)
**CAD (%)**	32 (37)	13 (36)	18 (41)	1 (14)	22 (37)	10 (36)
**Myocardial infarction (%)**	17 (20)	9 (25)	8 (18)	0 (0)	13 (22)	4 (14)
**LVEF <40%**	22 (25)	10 (28)	11 (25)	1 (14)	16 (27)	6 (21)
**CABG (%)**	7 (8)	4 (11)	3 (7)	0 (0)	5 (8)	2 (7)
**Serum sodium (mmol/l)**	137.8±0.5	137.1±0.9	138.6±0.5	136.5±1.0	138.1±0.4	137.3±0.5
**Serum potassium (mmol/I)**	4.1±0.1	4.1±0.1	4.1±0.1	4.3±0.3	4.0±0.1	4.3±0.4

BMI indicates body mass index, CAD indicates coronary artery disease, LVEF indicates left ventricular ejection fraction, CABG indicates coronary artery bypass grafting.

*p<0.05 vs. other variants.

Echocardiographic assessment revealed similar left atrial and ventricular diameters and left ventricular ejection fraction in all allele groups (Table 2). There was a trend towards larger diastolic interventricular septum diameter (IVSDD) in patients with Arg389Arg alleles (11.4±0.2 mm; Arg389Arg-Ser49Ser: 11.4±0.3 mm; Arg389Arg-Ser49Gly 11.3±0.3 mm) versus Arg389Gly (10.9±0.2 mm; Arg389Gly-Ser49Ser 11.2±3.8 mm; Arg389Gly-Ser49Gly 10.7±0.2 mm) and Gly389Gly alleles (10.1±0.3 mm; *p* = 0.05 between P389 polymorphisms). The SNP49 variants did not significantly modify the IVSDD. Cardiovascular medication was similar in groups with different polymorphisms (Table 3).

**Table 2:** Echocardiographic values in patients with different β_1_AR variants.

	All patients with AF N = 87	Arg389Arg N = 36	Arg389Gly N = 44	Gly389Gly N = 7	Ser49Ser N = 59	Ser49Gly N = 28
**LA (mm)**	46.7±0.8	47.6±1.5	46.0±1.1	47.1±1.4	47.6±1.0	44.7±1.5
**LVEDD (mm)**	51.5±0.7	51.4±0.9	52.0±0.9	49.4±2.5	50.7±0.7	53.3±1.3
**IVSDD (mm)**	11.1±0.2	11.4±0.2#	10.9±0.2	10.1±0.3	11.2±0.2	10.8±0.2
**LVEF (%)**	56.8±1.5	55.6±2.5	57.0±2.1	62.1±5.0	58.6±1.6	53.0±3.0

LA indicates left atrium, LVEDD indicates left ventricular enddiastolic diameter, IVSDD indicates diastolic interventricular septum diameter, LVEF indicates left ventricular ejection fraction.

**Table 3:** Cardiovascular medication in patients with different β_1_AR variants.

	All patients with AF N = 87	Arg389Arg N = 36	Arg389Gly N = 44	Gly389Gly N = 7	Ser49Ser N = 59	Ser49Gly N = 28
**Beta-blocker (%)**	79 (91)	34 (94)	39 (89)	6 (86)	53 (89)	26 (93)
**ACE-Inhibitor (%)**	27 (31)	12 (33)	13 (30)	2 (29)	19 (32)	8 (29)
**ARB (%)**	22 (25)	10 (28)	9 (20)	3 (43)	15 (25)	7 (25)
**Statin (%)**	38 (44)	17 (47)	19 (43)	2 (29)	25 (42)	13 (46)

ACE indicates angiotensin converting enzyme, ARB indicates angiotensin receptor blocker.

### β_1_AR Profile and Flecainide Success

**Figure 2:**
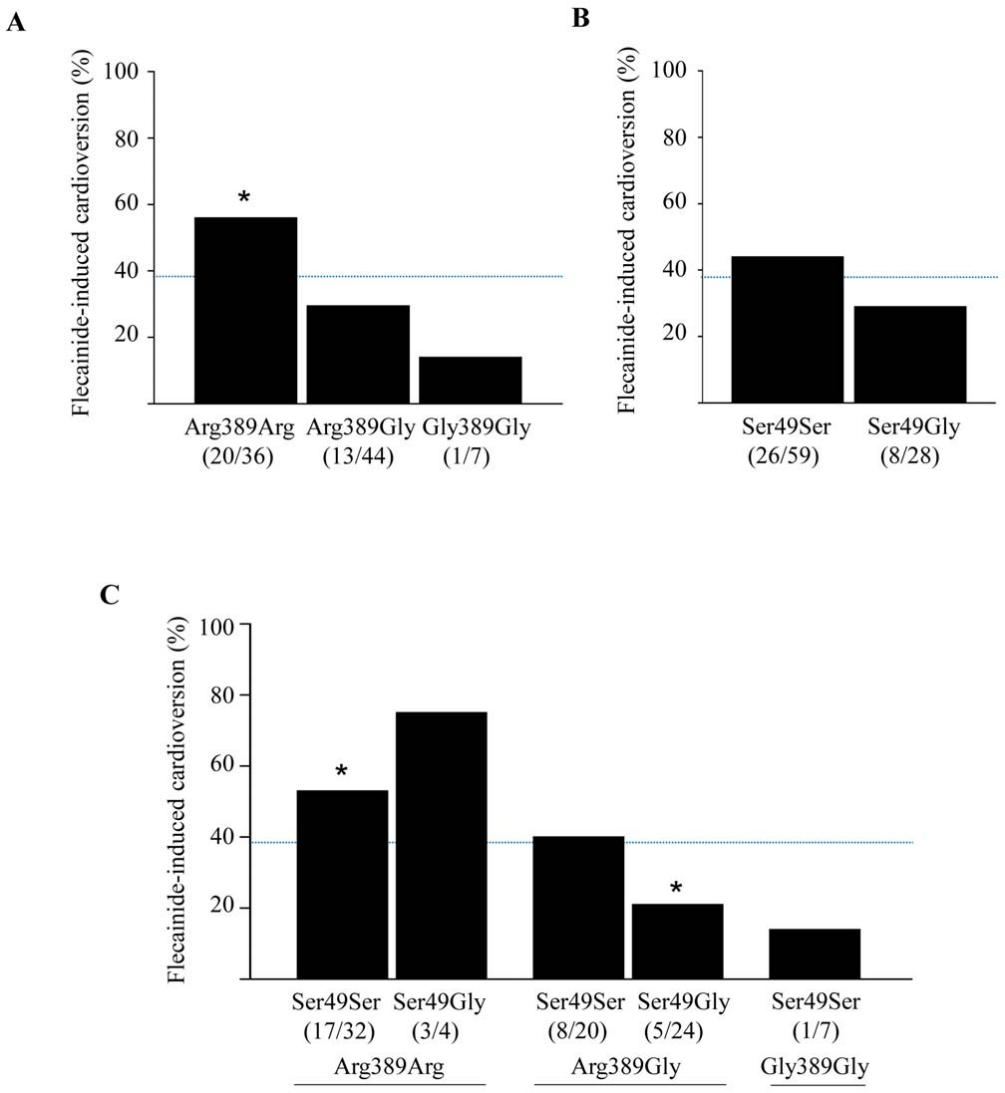
Success of flecainide-induced cardioversion in different β_1_AR genotypes, A, P389 polymorphism, B, P49 polymorphism, C, combination genotype of P389 and P49 variants. Dashed line marks the average cardioversion rate of 39%. *p<0.05 vs. other variants.

The overall cardioversion success rate after oral flecainide 300 mg was 39% (34/87). The significantly highest rate of sinus rhythm restoration of 55.5% (20/36) was found in patients homozygous to Arg389Arg polymorphism (OR 3.30; 95% CI; 1.34–8.13; *p* = 0.003; Figure 2).

In patients with Arg389Gly alleles flecainide was slightly less effective with a cardioversion rate of 29.5% (13/44; OR 0.44; 95% CI; 0.18–1.06; *p* = 0.066). In the small group of patients with Gly389Gly polymorphism in one of seven patients sinus rhythm could be restored (OR 0.24; 95% CI 0.03–2.07; *p* = 0.17). The SNP49 variants alone were not associated with the flecainide success in cardioversion. The combination of different SNP389 and SNP49 variants revealed the highest rate for a successful cardioversion with flecainide in patients with the genotype Arg389Arg independently to the coexisting SNP49 variants (Table 4, Figure 2). The lowest chance for a successful cardioversion was associated with the genotype Arg389Gly-Ser49Gly (OR 0.31; 95% CI 0.10–0.93; *p* = 0.03).

**Table 4:** Association between different genotypes and the success of flecainide-induced cardioversion.

	Flecainide Success	OR	95% CI	*p*
**Arg389Arg (n = 36)**	20 (55.5%)	3.30	1.34−8.13	0.003
Ser49Ser (n = 32)	17 (53.1%)	2.53	1.03−6.23	0.04
Ser49Gly (n = 4)	3 (75.0%)	5.03	0.50−50.52	0.14
Gly49Gly (n = 0)	-	-	-	-
**Arg389Gly (n = 44)**	13 (29.5%)	0.44	0.18−1.06	0.066
Ser49Ser (n = 20)	8 (40.0%)	1.05	0.38−2.92	0.93
Ser49Gly (n = 24)	5 (20.8%)	0.31	0.10−0.93	0.03
Gly49Gly (n = 0)	-	-	-	-
**Gly389Gly (n = 7)**	1 (14.3%)	0.24	0.03−2.07	0.17
Ser49Ser (n = 7)	1 (14.3%)	0.24	0.03−2.07	0.17
Ser49Gly (n = 0)	-	-	-	-
Gly49Gly (n = 0)	-	-	-	-

Mean heart rate of patients with successful cardioversion was during AF higher versus patients without sinus rhythm restoration, although statistical significance was not reached (108.0±4.9 bpm vs. 99.4±3.3 bpm; *p* = ns).

All patients in which pharmacological cardioversion failed underwent successful electrical cardioversion. At four weeks follow-up two patients showed a relapse of atrial fibrillation.

### β_1_AR Polymorphism and Risk of Atrial Fibrillation

To identify whether the risk for AF is linked to a specific β_1_AR polymorphism, we compared the relative allele frequencies in patients with AF (AF-group; n = 87) to patients with no history of AF (control-group; n = 58).

**Figure 3:**
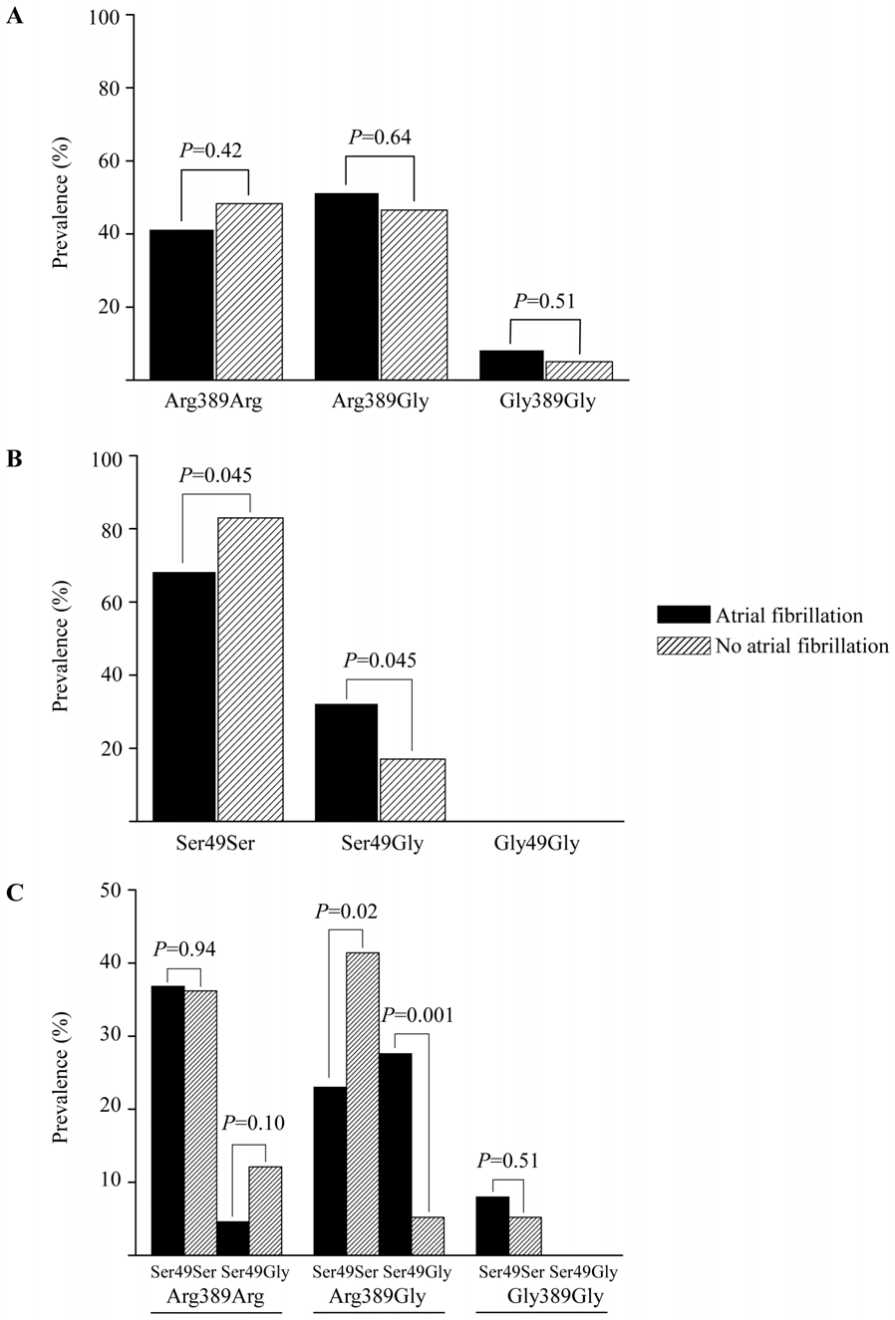
Relative allele distribution in patients with and without AF. A, P389 variants, B, P49 variants, C, combined genotype of P389 and P49 variants.

This comparison revealed quite similar SNP389 allele distributions in patients with AF versus control-group (Arg389Arg: 41.4% AF-group vs. 48.3% control-group, *p* = ns; Arg389Gly: 50.6% AF-group vs. 46.6% control-group, *p* = ns; Gly389Gly: 8.0% AF-group vs. 5.1%, *p* = ns; Figure 3A). The prevalence of the Ser49Ser polymorphism was tendentiously higher in patients without AF versus those with AF (32.2% vs. 17.2%; *p* = 0.045; Figure 3B). Conversely, Ser49Gly genotype was slightly more common in patients with than without AF (73.7% vs. 26.3%; *p* = 0.045). The combination of both SNPs revealed that the prevalence of the Arg389Gly-Ser49Gly genotype was significantly higher in patients with versus without AF (27.6% versus 5.2%; *p* = 0.001; Figure 3C). Thus, a hazard ratio of 6.98 for AF in patients with the Arg389Gly-Ser49Gly genotype was calculated (95% CI, 1.99–24.46; *p*<0.001).

## Discussion

The Arg389Gly polymorphism has been shown to be associated with heart failure, acute myocardial infarction, hypertension and left ventricular remodeling in response to beta- blockade [19], [20], [21].

**Figure 4:**
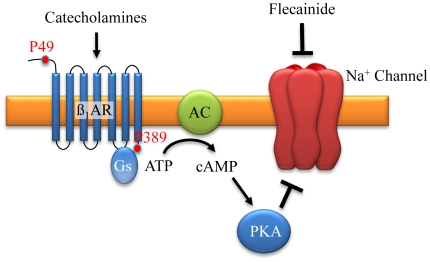
Schematic explanation of beta_1_-adrenoceptor and flecainide interaction. Localisation of P49 on N-terminus and P389 on C-terminus. β_1_AR activation results via G protein (Gs) and adenylate cyclase (AC) pathway in increase of cyclic adenosine monophosphate (cAMP) and activation of protein kinase A (PKA). PKA induced phosphorylation inactivates I_Na_ channel activity. Flecainide inhibits I_Na_ directly.

We found that the Arg389Arg polymorphism was strongly associated with a higher efficiency of flecainide action in patients with AF. Reported different affinity of β_1_AR SNP389 variants to catecholamines may explain the underlying mechanism of this observation [11], [22], [23]. In vitro experiments have demonstrated that the Arg389Arg genotype is a gain-of-function variant with enhanced coupling to Gs protein, following a slightly elevated basal and three- to four-fold higher adenylate cyclase activity than Gly389 variants [10]. Isoproterenol-stimulation enhances Gs protein activation and adrenergic-mediated reduction of sodium current [24]. This accessory reduction of sodium influx may potentiate the flecainide induced-blockade of I_Na_ (Figure 4).

Compared to more healthy subjects we noticed a poorer cardioversion rate in population with organic heart diseases despite the expected higher susceptibility to flecainide due to elevated catecholamine concentrations in these patients [1], [2], [25], [26]. But contrarily, diseased cardiomyocytes promote AF due to complex electrical and structural remodeling [27], [28], [29], [30]. These changes may outbalance the supposed increased antiarrhythmic effects of flecainide. Chronic treatment with flecainide has been reported to be harmful, particularly in patients with structural heart diseases and myocardial infarction [31]. Higher serum catecholamine concentrations in patients with cardiovascular diseases may have augmented the β_1_AR-induced I_Na_ blockade and the proarrhythmic effects of flecainide, especially in patients with vulnerable myocardium and the Arg389Arg genotype [25], [26], [32], [33], [34], [35].

Newer antiarrhythmic agents like vernakalant and dronedarone with sodium channel blocking attribute may be similarly influenced by β_1_AR variants [36], [37].

Resting heart rate has been associated with Arg389Gly variants [38], [39]. Concordantly, we observed during AF significantly higher ventricular heart rates in homozygous patients for the arginine allele and the lowest heart rate in patients homozygous for the glycine variant (heart rate Arg389Arg>Arg389Gly>Gly389Gly). This may have clinical consequences and patients with Arg389Arg genotype may need intensified beta-blocker treatment.

The interventricular septum thickness was larger in patients with Arg389Arg than in carriers of other variants. This may be explained due to elevated catecholamine impact on cardiomyocytes in this genotype and enhanced hypertrophy signaling [40]. The β_1_AR SNP49 polymorphism itself was not associated with functional effects of flecainide. But in combination with the P389 polymorphism flecainide was most effective in Arg389Arg-Ser49Ser genotype and least effective in Arg389Gly-Ser49Gly genotype. This finding is supported by previous reports that the SNP49 variants have not an effect on the adenyl cyclase activity and position 389, but not 49, obviously determines the functional responsiveness of β_1_AR, and P49 may only play a modulating role [9]. The genotype Arg389Arg-Ser49Gly may need higher flecainide concentrations for successful cardioversion.

In the present study the SNP389 β_1_AR genotype alone was not, and the Ser49Gly genotype slightly associated with the AF prevalence. But in combination of both SNPs, the genotype Arg389Gly-Ser49Gly was associated with an almost 7-fold higher risk for AF than other genotypes. Despite the limited sample size in our cohort, this finding confirms a recent report that the Ser49Gly variant may be associated with AF [41]. Further studies are needed to figure out the causal explanation for this supposed genetic determination of AF.

The present investigation provides more insight into the potential influences of β_1_AR polymorphism on antiarrhythmic drug action, which are currently underestimated. Our results indicate that genetic testing might help identifying patients at elevated risk for AF. When this observation is confirmed in larger cohorts, preventive strategies in these patients might be helpful. Further studies with genetic testing may help to identify patients with organic heart diseases in whom a differential antiarrhythmic therapy might be indicated. Newer antiarrhythmic drugs may also interfere with the β_1_AR polymorphism.
